# Development of a cost‐effective high‐throughput mid‐density 5K genotyping assay for germplasm characterization and breeding in groundnut

**DOI:** 10.1002/tpg2.70019

**Published:** 2025-03-31

**Authors:** Manish K. Pandey, Vinay Sharma, Aamir W. Khan, Pushpesh Joshi, Sunil S. Gangurde, Prasad Bajaj, Pasupuleti Janila, Annapurna Chitikineni, Ramesh Bhat, Babu N. Motagi, Chandramohan Sangh, Thankappan Radhakrishnan, Sandip K. Bera, Gregor Gorjanc, Krishna Reddy Gujjula, Nathan Hall, Claudio D. Carrasco, Kandalam Arjun, Srinivas Chandram, Rajeev K. Varshney

**Affiliations:** ^1^ Center for Excellence in Genomics and Systems Biology (CEGSB) and Center for Pre ‐Breeding Research (CPBR) International Crops Research Institute for the Semi‐Arid Tropics (ICRISAT) Hyderabad India; ^2^ Department of Biotechnology University of Agricultural Sciences Dharwad India; ^3^ ICAR‐Indian Institute of Groundnut Research (IIGR) Junagadh India; ^4^ The Roslin Institute and The Royal (Dick) School of Veterinary Studies University of Edinburgh Edinburgh UK; ^5^ Thermo Fisher Scientific Waltham Massachusetts USA; ^6^ WA State Agricultural Biotechnology Centre, Centre for Crop and Food Innovation Murdoch University Murdoch WA Australia

## Abstract

Groundnut (*Arachis hypogaea* L.), also known as peanut, is an allotetraploid legume crop composed of two different progenitor sub‐genomes. This crop is an important source for food, feed, and confectioneries. Leveraging translational genomics research has expedited the precision and speed in making selections of progenies in several crops through either marker‐assisted selection or genomic selection, including groundnut. The availability of foundational genomic resources such as reference genomes for diploid progenitors and cultivated tetraploids, offered substantial opportunities for genomic interventions, including the development of genotyping assays. Here, a cost‐effective and high‐throughput genotyping assay has been developed with 5,081 single nucleotide polymorphisms (SNPs) referred to as “mid‐density assay.” This multi‐purpose assay includes 5,000 highly informative SNPs selected based on higher polymorphism information content (PIC) from our previously developed high‐density “Axiom_*Arachis*” array containing 58,233 SNPs. Additionally 82 SNPs associated with five resilience and quality traits were included for marker‐assisted selection. To test the utility of the mid‐density genotyping (MDG) assay, 2,573 genotypes from distinct sets of breeding populations were genotyped with the 5,081 SNPs. PIC of the SNPs in the MDG ranged from 0.34 to 0.37 among diverse sets. The first three principal components collectively explained 82.08% of the variance among these genotypes. The mid‐density assay demonstrated a proficient ability to distinguish between the genotypes, offering a high level of genome‐wide nucleotide diversity. This assay holds promise for possible deployment in the identification of varietal seed mixtures, genetic purity within gene bank germplasms and seed systems, foreground and background selection in backcross breeding programs, genomic selection, and sparse trait mapping studies in groundnut.

## Core Ideas

- Developed a mid‐density genotyping assay with 5,081 highly informative SNPs.
- A total of 82 trait‐associated SNPs were also included in this assay for foreground selection.
- A comprehensive diversity analysis study was performed using mid‐density assay on 2,573 genotypes.
- The efficiency of mid‐density (5K) assay was tested for foreground and background selection and compared with high density.

**Abbreviations**
ABLs: advanced breeding lines
FP: founder parents
GBS: genotyping‐by‐sequencing
MABC: marker‐assisted backcrossing
MAF: minor allele frequency
MAS: marker‐assisted selection
MDG: mid‐density genotyping
PCA: principal component analysis
PIC: polymorphism information content
QC: quality control
QTL: quantitative trait locus
RS: reference set
RV: released varieties
SNP: single nucleotide polymorphism
TASSEL: Trait Analysis by Association, Evolution, and Linkage
T‐GBS: targeted genotyping‐by‐sequencing
TP: training population
TVC: torrent variant caller

## 1 INTRODUCTION

Groundnut (*Arachis hypogaea* L.) is an allotetraploid crop, composed of two progenitor sub‐genomes, with chromosome number 2*n* = 2*x* = 20 and genome size of ∼2.7 Gb for cultivated groundnut. Groundnut is an important source of protein, oil, and minerals cultivated in more than 100 countries worldwide. Notably, groundnut improvement aims to increase productivity, nutritional value, and resilience against biotic and abiotic stresses. Translational genomics expedited selection process with precision in several crops, including groundnut, through either marker‐assisted selection (MAS) (Bera et al., 2018, 2019; Janila et al., 2016; Kolekar et al., 2017; Pandey et al., 2024; Shasidhar et al., 2020; Varshney et al., 2014; Yeri & Bhat, 2016) or genomic selection (Pandey et al., 2020). The development of high‐quality reference genomes for both diploid progenitor species (*A. duranensis* & *A. ipaensis*) and the cultivated subspecies (subsp. *hypogaea* and subsp. *fastigiata*) of tetraploid groundnut mark as an important milestone in groundnut genomics (Bertioli et al., 2016; X. Chen et al., 2016; Bertioli et al., 2019; X. Chen et al., 2019; Zhuang et al., 2019).

Various genotyping assays, with varied marker densities, are needed based on the scale and type of application in a breeding program. While whole‐genome sequencing with varied depth and coverage provides one of the most flexible and informative options for genotyping, it is not cost‐effective. Furthermore, the requirement of sound technical expertise to analyze sequencing data hinders the deployment of large‐scale genotyping of samples in gene bank, breeding, and seed systems. High‐density genotyping (HDG) arrays, such as single nucleotide polymorphism (SNP) arrays (ranging from 10,000 SNPs to 250,000 SNPs), are available for multiple crops. Due to substantial similarity between the sub‐genomes of tetraploid groundnut, an HDG platform was designed for genetic diversity and trait mapping studies in groundnut. Specifically, International Crops Research Institute for the Semi‐Arid Tropics (ICRISAT) led the development of a HDG array (Axiom_ *Arachis* Array) with 58K genome‐wide SNPs in collaboration with the University of Georgia and Thermo Fisher Scientific (Pandey et al., 2017). So far, the international groundnut research community has used HDG array in >25 studies of genetic diversity, trait mapping, and background genome recovery in backcross breeding (Pandey et al., 2020; Shasidhar et al., 2020).

While the Axiom_*Arachis* array is sufficient for studies related to genetic diversity and trait mapping through genetic and association analysis, it is not cost‐effective for large‐scale application in groundnut breeding. Therefore, a cost‐effective mid‐density genotyping (MDG) assay is necessary with around 2,000–6,000 SNPs to be deployed in large‐scale applications with reduced per sample cost of about 10 USD. HDG arrays, typically consisting of 50K–80K of SNPs, are significantly more expensive, with costs ranging from 50 to 60 USD per sample, including DNA extraction, depending on the platform. MDG assay can be used in genetic diversity, background genome recovery, genomic selection, genetic purity, and tracking linkage drag in wide crosses.

MDG is an established tool for large‐scale adoption in breeding programs. It is desirable to keep MDG assay dynamic so that the useful SNPs from genomics research can be incorporated, making the MDG assay more and more informative over time for breeders and researchers. Recently, a new MDG assay with 4,000 SNPs has been developed in pearl millet using the AgriSeq platform with targeted genotyping‐by‐sequencing (T‐GBS) (Semalaiyappan et al., 2023). Here, we developed and validated a new cost‐effective MDG assay in groundnut with 5,081 SNP markers for routine application of genomics in genetic diversity, background genome recovery in recurrent parents, checking duplications and genetic mixture, and most importantly, genomic selection. The AgriSeq T‐GBS assay was developed with 5,081 SNPs including trait‐associated SNPs for seven to eight resilience and quality traits in groundnut. We are deploying this assay for large‐scale genomic selection in groundnut at ICRISAT and National Agricultural Research System (NARS) locations. This assay will enable widespread adoption in several other new applications in addition to genetic diversity and trait mapping, background selection, and genomic selection, thereby contributing to the advancement of future groundnut breeding programs.

**Core Ideas**

- Developed a mid‐density genotyping assay with 5,081 highly informative SNPs.
- A total of 82 trait‐associated SNPs were also included in this assay for foreground selection.
- A comprehensive diversity analysis study was performed using mid‐density assay on 2,573 genotypes.
- The efficiency of mid‐density (5K) assay was tested for foreground and background selection and compared with high density.

## 2 MATERIALS AND METHODS

### 2.1 Sources for SNPs and selection of best set of informative SNPs

**FIGURE 1:**
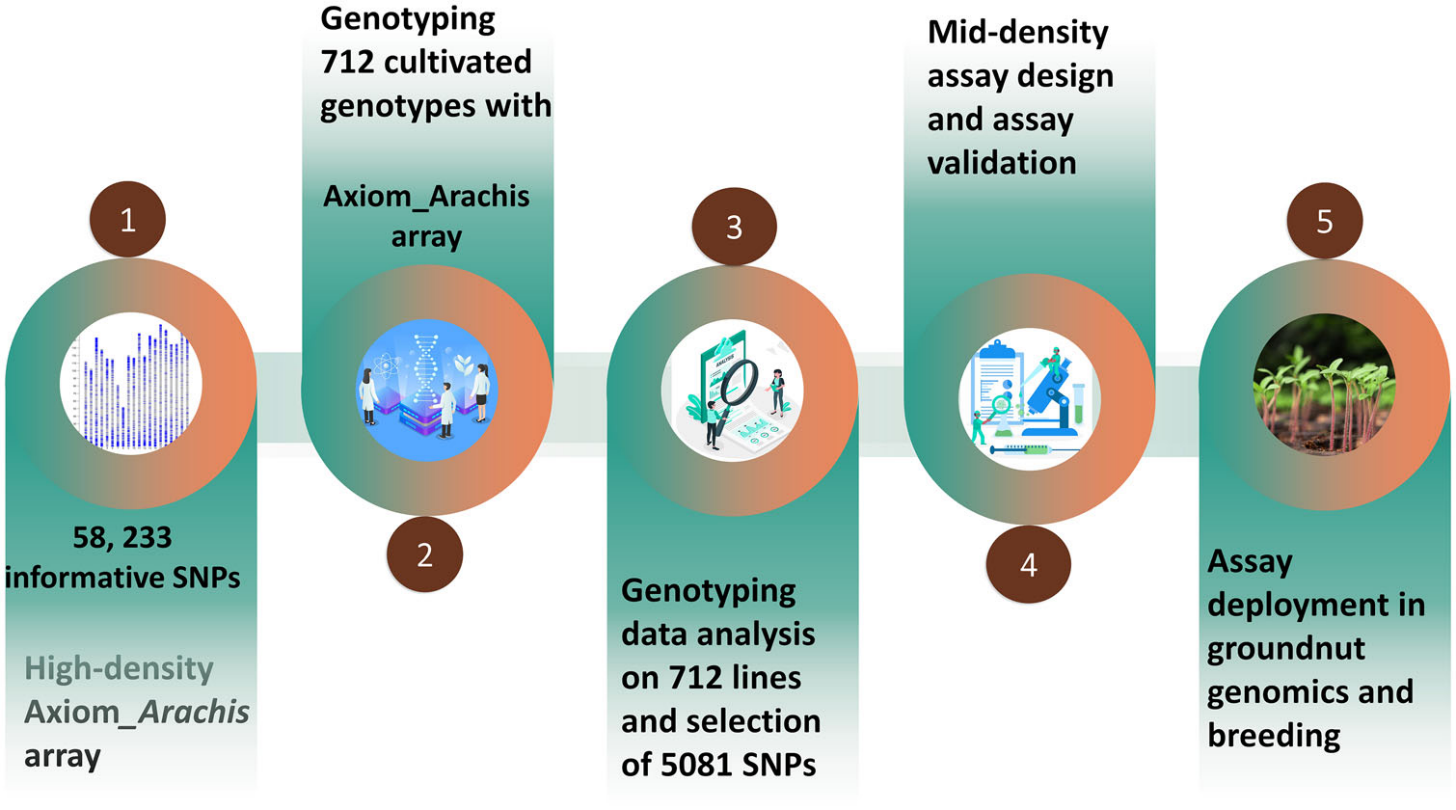
Flowchart for developing mid‐density genotyping assay in groundnut with 5,081 informative single nucleotide polymorphisms (SNPs).

The genotyping data were generated using the high‐density Axiom_*Arachis* array, containing 58,233 unique and informative SNPs (Pandey et al., 2017) on a diverse panel of 722 cultivated groundnut genotypes, which had 254 from the ICRISAT reference set (RS), 285 from the ICRISAT training population (TP), and 174 released varieties (RV) across India. The seven genotypes were common to the RS and TP. We analyzed these data and selected 8,200 high‐quality SNPs based on the criteria of high polymorphism information content (PIC; between 0.374 and above) and uniform genome coverage. Previous studies also reported SNP markers with similar PIC values as informative for genetic diversity and population structure analysis (H. Chen et al., 2011; Garcia et al., 2018; Singh et al., 2013). All the SNPs were initially subjected to check for missing calls <0.2, and those with heterozygous calls >0.05 (>5% heterozygous calls) were removed. Next, priority was given to SNPs that did not have another SNP within the flanking region of 200 bp. Finally, a total of 82 trait‐associated SNPs identified from different studies were included, such as those associated with resistance to rust and late leaf spot, high oleic acid, seed weight, fresh seed dormancy, and shelling percentage (Figure 1).

### 2.2 Assay design and validation

The AgriSeq T‐GBS assay design comprises three sequential steps, such as (1) pre‐design quality control (QC), (2) AgriSeq Primer designs, (3) post‐design QC, and (4) final assay development. Initially, ∼8,027 SNP markers were selected and further filtered for the assay. Using the “Tifrunner” reference genome (Bertioli et al., 2019), the uniqueness of the context sequence for each candidate marker was examined by mapping it to the reference genome.

Since the 8,027 SNPs were initially identified on diploid progenitor genomes (Bertioli et al., 2016) for the development of a 58K Axiom_*Arachis* array (Pandey et al., 2017), additional computational work was undertaken to identify marker position in the tetraploid reference genome, Tifrunner (Bertioli et al., 2019). Each marker was mapped to an individual sub‐genome separately to identify the location of the marker with the mapping threshold of 95%. For each marker, results of mapping on separate sub‐genomes were classified into (a) mapping uniquely and the marker position is identified, (b) mapping uniquely, with region identified but the marker position is not identified, (c) mapping to multiple locations on the same sub‐genome, and last (d) not mapping to any sub‐genome.

Based on the mapping results against the reference genome, markers were categorized as follows: (a) unique to a particular sub‐genome (A or B); (b) mapping to two loci (one each in sub‐genome A and B); and (c) mapping to more than two loci on the reference genome or mapping to exactly two loci on the same sub‐genome (A or B). These markers were then divided into three categories. In Category 1, the marker sequence maps uniquely to one of the sub‐genomes, while the Category 2 marker sequence maps to two loci on the genome (one on sub‐genome A and another on sub‐genome B), and in Category 3 marker sequence maps to two loci on the genome, either both on sub‐genome A or both on sub‐genome B or maps to more than two loci. The primer designing for Category 3 markers (associated with traits) was removed from the design process at this stage and primer design was done separately.

**FIGURE 2:**
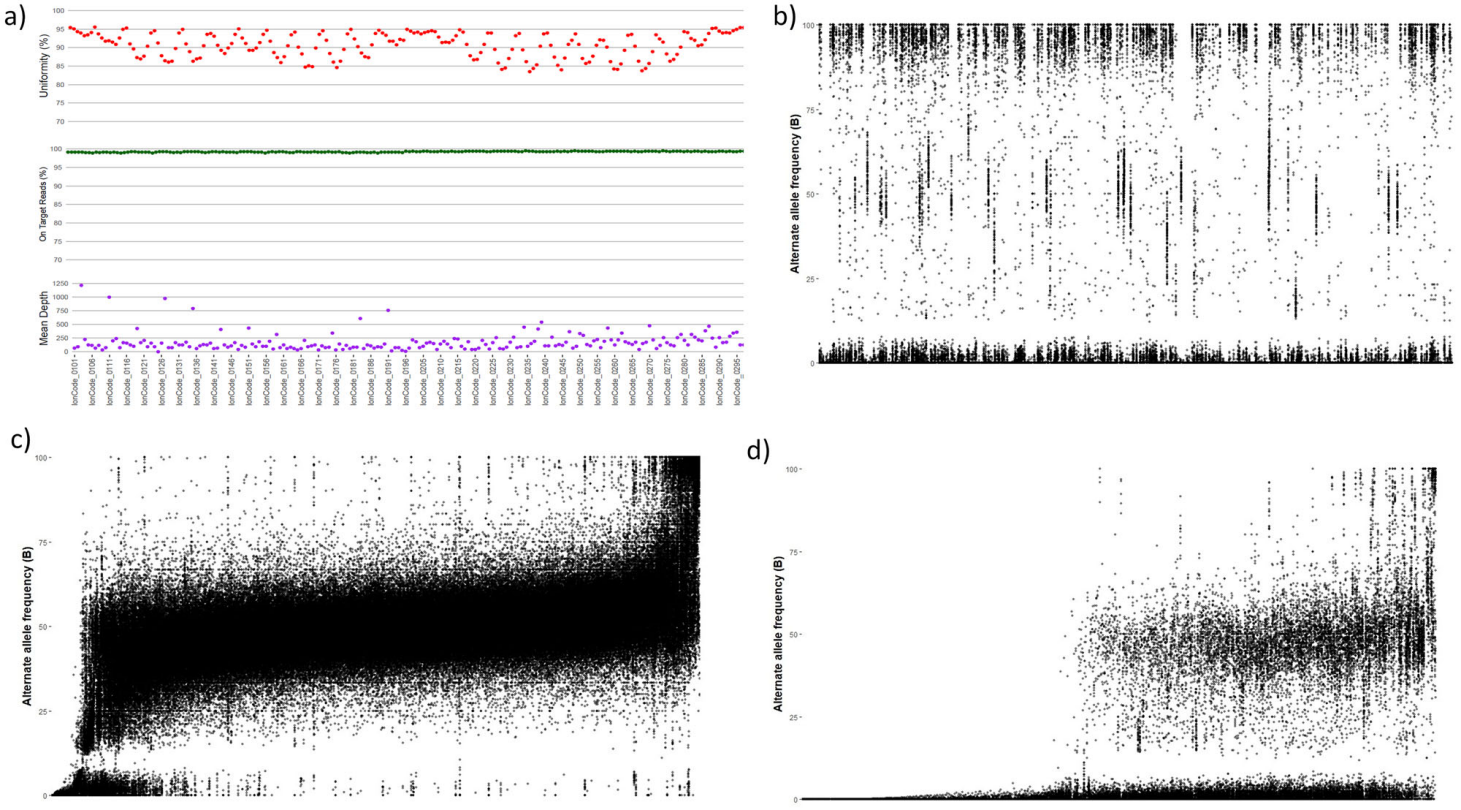
Quality matrix for validation samples and call types for markers from progenitor diploid and cultivated tetraploid reference genome. (a) Quality metrics for 192 samples (>20X coverage); (b) total 963 markers from progenitor diploid genome expected allele frequencies of 0.0, 0.5, 1 for AA, AB, BB, respectively; (c) total 3393 markers from tetraploid genome with expected allele frequencies of 0.5, 0.75, 1 for AABB, ABBB, BBBB, respectively; (d) total 669 markers from tetraploid genome expected allele frequencies of 0.5, 0.75, 1 for AAAA, ABAA, BBAA, respectively.

The primers were designed for Category 1 and 2 markers. The designer pipeline runs iteratively, modifying primer design and amplicon parameters (e.g., GC [guanine and cytosine] content and Tm [melting temperature] values) sequentially to find all possible amplicons, which consist of primer pairs and an insertion sequence, for each marker. The designer pipeline chooses the best amplicon that contains the marker. During this process, the specificity of the primers was checked on the reference genome. Some of the primers were discarded due to specificity. Post‐design quality check was performed for Category 1 markers with primer designs amplifying one sub‐genome (either A or B), while Category 2 markers with primer designs amplifying both sub‐genomes (A and B) (Figure 2). After filtering, approximately 5,000 SNPs were finalized from the initially selected set of 8,027 SNPs. Additionally, 82 highly informative SNPs were finalized from a subset of 213 trait‐associated SNPs. In total, 5,081 highly informative SNPs were used for assay design and validation.

### 2.3 Assay validation

The AgriSeq T‐GBS workflow starts with sample concentration, initially checked using the Quant‐IT Ds DNA assay (Q32851, Invitrogen), followed by sample dilutions for normalization of uniform DNA concentrations. Subsequently, 3 µL of normalized DNA was used for library amplification, employing AgriSeq HTS library kit (A34143, Life Technologies) followed by 16 min amp/cycles (12 cycles) and 20 min pre‐ligation of IonCode Barcode Adapters (IonCode_0101 to IonCode_0296). Genotyping was performed using the Ion 550 Chef kit (A43542‐Life Technologies) and Ion 550 chip (∼120 million reads) (A42962‐Life Technologies). This assay was validated on a set of 386 diverse lines.

**FIGURE 3:**
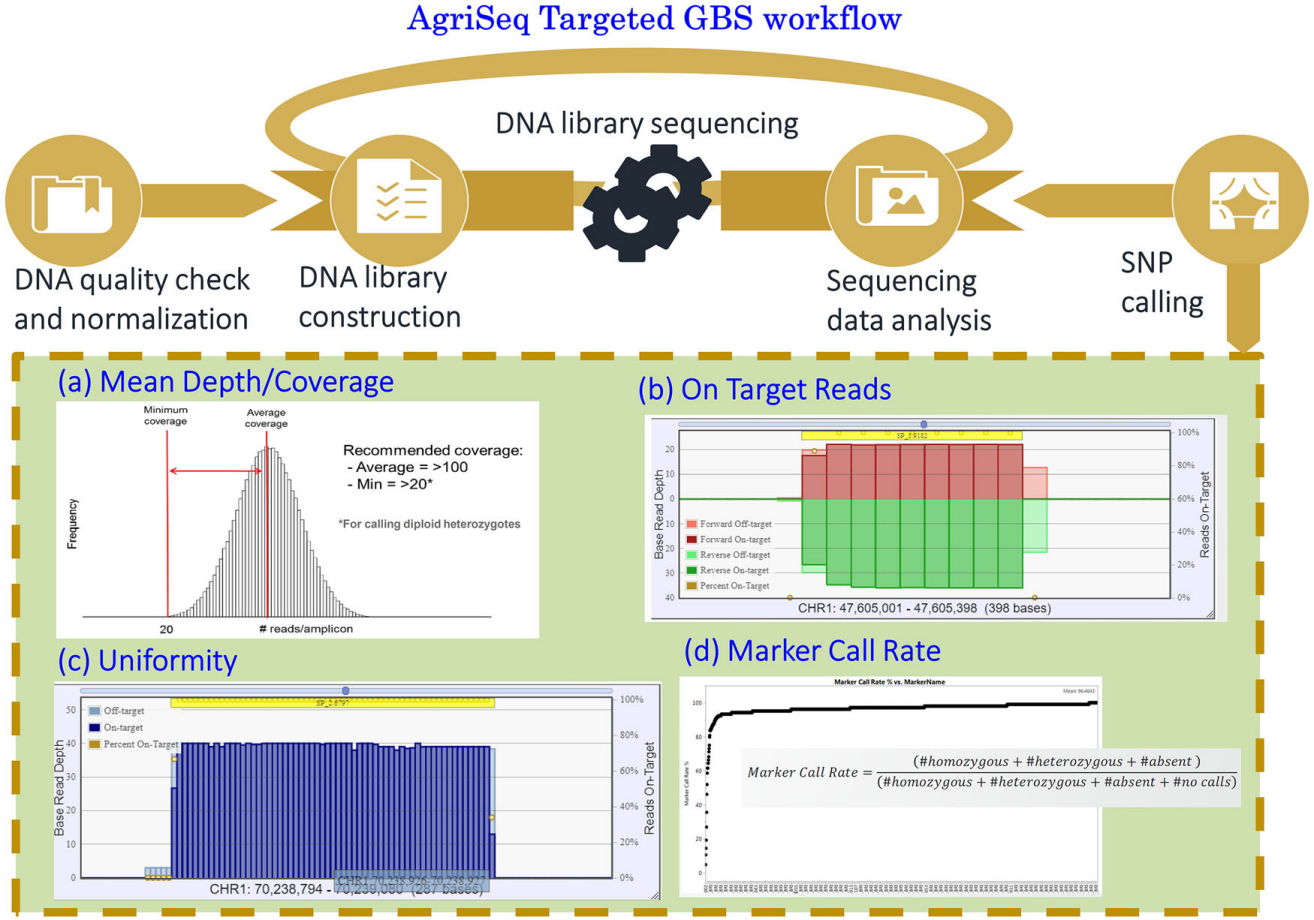
Workflow for groundnut AgriSeq targeted genotyping‐by‐sequencing‐based mid‐density assay genotyping. GBS, genotyping‐by‐sequencing.

To ensure high‐quality genotyping‐by‐sequencing (GBS), the number of reads per amplicon was monitored during sequencing, aiming for a mean coverage depth of at least 100X to increase the likelihood that most markers having a minimum coverage of 5X. The attention was given to assessing the percentage of mapped reads and ensuring that on‐target reads aligned correctly over a target region, with a focus on achieving metric values that minimize off‐target alignments, indicating potential issues such as sample contamination and uncharacterized genetic variation. Note that the low uniformity indicates poor sequencing across the entire amplicon. Therefore, the uniformity was maintained by ensuring a percentage of target bases with at least 0.2 times the mean depth. This feature is a measure of end‐to‐end coverage over the target region. Finally, the Marker Call Rate was assessed by calculating the percentage of all samples generating a genotype call for a specific marker. The emphasis was given to designing assays with high‐performing markers, targeting an overall mean marker call rates >90% (Figure 3).

### 2.4 Genotyping

Genotyping was performed using Torrent Variant Caller (TVC) for 963 category‐1 markers and AgriSeqPoly and 4,118 category‐2 markers. TVC is suitable for diploid markers and AgriSeqPoly is utilized to genotype both allo‐ and auto‐polyploid markers. The sequenced reads were de‐multiplexed to individual samples using the barcode sequences. For each sample, the sequenced reads from the targeted regions were mapped to the reference genome using Torrent Mapping Alignment Program (https://github.com/iontorrent/TMAP), followed by genotyping using TVC (https://github.com/iontorrent/Torrent‐Variant‐Caller‐stable). Both TVC and AgriSeqPoly implement a Bayesian model, using the number of reads supporting each allele as input to output a genotype call.

### 2.5 Assay deployment for performing genetic diversity in a set of 2,573 genotypes

MDG assay was used for genotyping 1776 genotypes on the AgriSeq T‐GBS platform. This diverse set included 270 founder parents (FP) from breeding programs (ICRISAT, UAS‐Dharwad, and ICAR‐DGR), along with 1,506 advanced breeding lines (ABLs). Additionally, SNP calls for 797 genotypes were extracted from genotyping data generated using the high‐density Axiom_*Arachis* array, comprising 297 RSs, 294 TPs, and 206 RV. The genotyping data from this set of 2,573 genotypes were then analyzed to assess genetic diversity.

Genotyping data were filtered before being uploaded into Trait Analysis by Association, Evolution, and Linkage (TASSEL) v5.0 (Bradbury et al., 2007), which was used to further filter samples and sites, and produce a dendrogram of samples (https://www.maizegenetics.net/tassel). Thorough filtering helped to exclude uncalled sites, enhancing the accuracy of the predicted tree. Before uploading data to TASSEL, filtering was performed and the ambiguous SNP IDs and associated calls were identified and removed, considering instances where two or more IDs occurred at the same position or when one ID had two or more positions associated with it. Sample names were converted to a standardized format compatible with downstream analyses, and a conversion table was created. The conversion table associates the original sample name with the new standardized name. All samples were added to a single data matrix, saved as a comma‐separated value file, and modified using Microsoft Excel to be a TASSEL‐compatible hapmap format. Positions with multi‐SNP calls were excluded, as TASSEL does not process multi‐SNP data. Following filtration, the data file was loaded into TASSEL for further filtration using three approaches (unfiltered, less strict, and strict). The stringent dataset was generated by filtering sites to include only loci with a ≥2,000 calls, followed by retaining samples with a proportion of 0.95 of sites present, resulting in 2153 samples with 4714 sites. Alternatively, the less stringent dataset was generated by filtering loci with calls of ≥2,700, retaining only samples with a proportion of 0.90 sites present, resulting in 2,639 samples with 2,988 sites. The resulting datasets were used for subsequent analysis. For each dataset, a distance matrix was constructed, and multidimensional scaling (MDS), similar to a principal component analysis (PCA), was performed using TASSEL. Neighbor‐joining trees were constructed for the less strict and strict datasets. Attempts to create a tree from the unfiltered dataset yielded no informative result. For data visualizations, MDS outputs were visualized with Plotly in Python 3.9 and saved as an interactive graphic embedded in an html document. Additionally, trees were rendered using ete3 in Python 3.9 and saved as scalable vector graphics for comprehensive visualization and analysis.

## 3 RESULTS

### 3.1 SNP selection and assay design

Previously, we developed an HDG array containing 58,233 unique and informative SNPs (Pandey et al., 2017) and used it to genotype 722 cultivated groundnut genotypes (Table S1). This diverse panel comprised accessions from the ICRISAT RS (254 cultivated accessions), the ICRISAT TP (285 accessions), and RV across India (174 varieties), with seven common genotypes shared across the three panels. Based on the PIC and uniform genome coverage, 8,027 SNPs were selected, with a PIC value of 0.374 and above (Table S2). Of these, 5,000 SNPs were prioritized, while the remaining 3,027 were kept in reserve, considering their PIC and genome coverage. Further, as an essential requirement for this platform, priority was given to only those SNPs that did not have any another SNP within flanking region of 200 bp. The primer sequence was analyzed using blast analysis against the reference genome for priority one markers, 863 markers mapped with zero mismatche and uniquely to one sub‐genome of Tifrunner, 3,991 markers mapped with zero mismatches but to two locations (one on sub‐genome A and another on sub‐genome B), and the remaining 1099 designs/primers have mismatches with the Tifrunner reference.

In addition to 8,027 SNPs, a total of 82 traits associated with SNPs from different studies were included, covering traits such as resistance to rust and late leaf spot, high oleic acid, seed weight, fresh seed dormancy, and shelling percentage. These 82 SNPs were identified through trait mapping using reference genomes (Table S3), specifically 97 SNPs (58 Category 1, 25 Category 2, and 14 Category 3) from *A. duranensis* (Bertioli et al., 2016), 104 (47 Category 1, 18 Category 2, and 39 Category 3) from Shitouqi (Zhuang et al., 2019), and 12 (6 Category 1, 1 Category 2, and 5 Category 3) from Tiffrunner (Bertioli et al., 2019) assembly. A similar analysis was performed for these markers, which showed 111 SNPs as Category 1, 44 SNPs as Category 2 markers, and 58 Category 3 markers.

**FIGURE 4:**
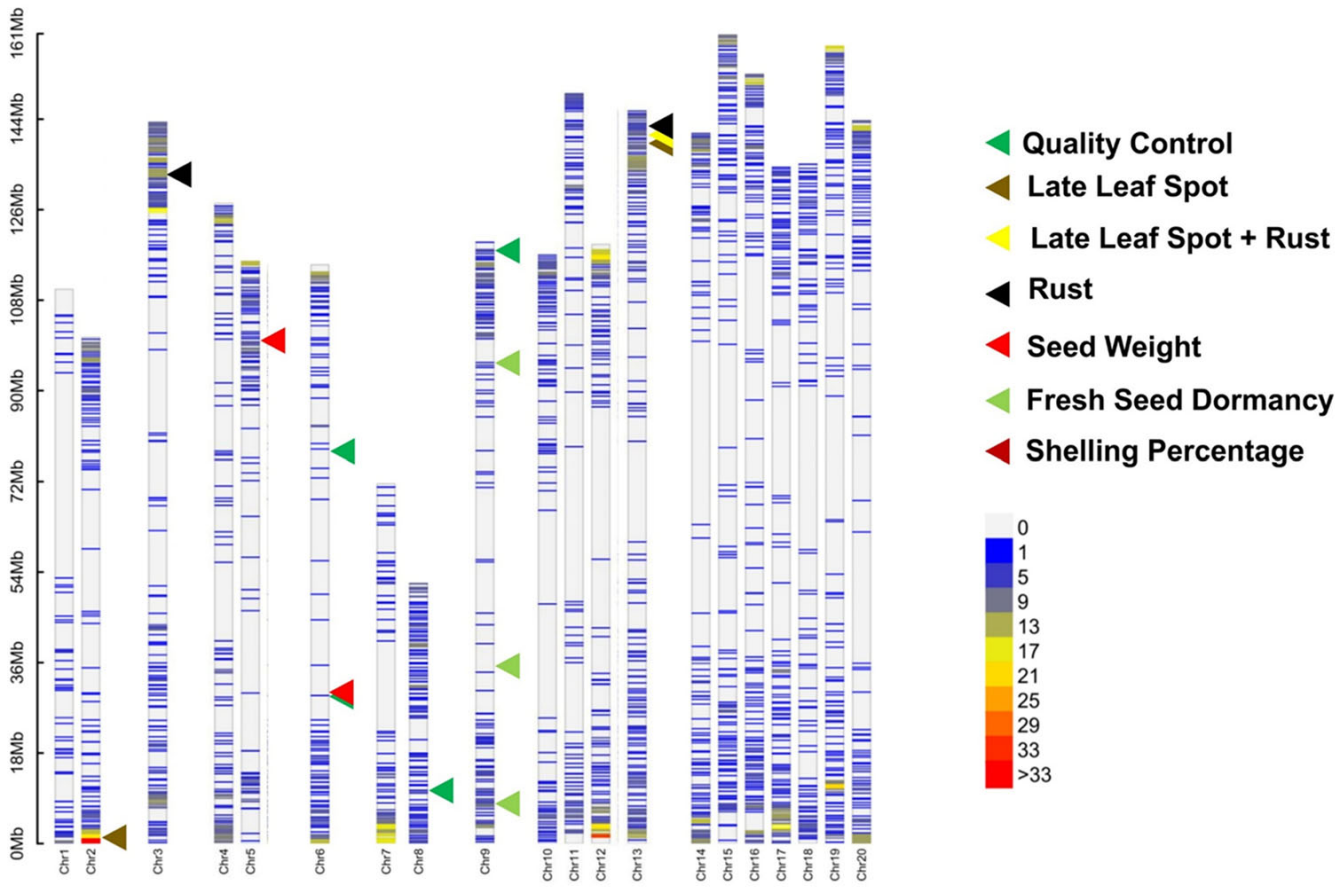
Genome‐wide distribution of single nucleotide polymorphism (SNP) markers on mid‐density genotyping assay on 20 chromosomes of cultivated groundnut genome. The triangles indicate the SNP positions of trait‐associated SNPs for five quality‐ and resilience‐related traits as well as 20 quality control (QC) SNPs.

The final assay comprised 5,081 SNPs, consisting of 963 Category 1 markers (amplifying either A or B sub‐genome) and 4,118 Category 2 markers (amplifying both sub‐genome A and B) (Table S4). In total, the final assay included 5,063 SNPs, eight deletions, and 10 insertions. Of the total 5,081 SNP markers, 2,428 SNP markers were selected from A sub‐genome (Chromosomes 1–10), and 2,653 SNP markers from B sub‐genome (Chromosomes 11–20) (Table 1) (Figure 4). The distribution of markers across chromosomes varied, from 187 (Chromosome 17) to 393 (Chromosome 3), with an average of 254 SNP markers per chromosome.

**TABLE 1:** Number of single nucleotide polymorphism (SNP) markers from different chromosomes in mid‐density genotyping assay.

Chromosome	Number of markers	Chromosome	Number of markers
Arahy.01	264	Arahy.11	211
Arahy.02	278	Arahy.12	347
Arahy.03	393	Arahy.13	338
Arahy.04	227	Arahy.14	272
Arahy.05	225	Arahy.15	237
Arahy.06	216	Arahy.16	281
Arahy.07	196	Arahy.17	261
Arahy.08	196	Arahy.18	187
Arahy.09	252	Arahy.19	285
Arahy.10	181	Arahy.20	234
Sub‐genome A	2428	Sub‐genome B	2653
Total markers on the groundnut Agriseq mid‐density genotyping assay			5081

### 3.2 Verification and validation of new mid‐density genotyping assay

**FIGURE 5:**
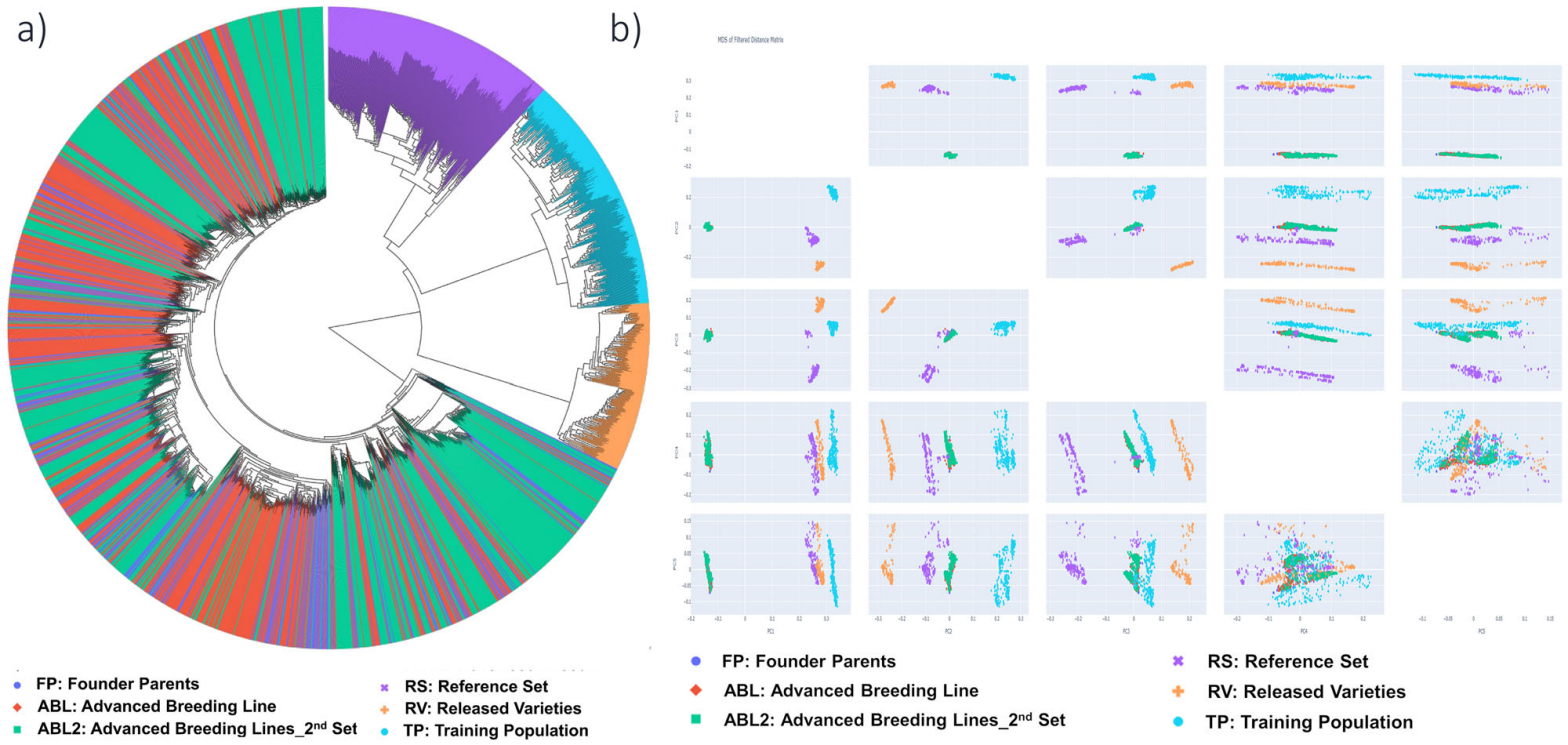
Deployment of mid‐density assay for genetic diversity analysis of a diverse set containing genotypes from founder parents (FP) of ICRISAT breeding program, training population (TP), ICRISAT reference set (RS), two sets of advanced breeding lines (ABLs), and released varieties (RV).

The high‐quality GBS matrix was ensured during sequencing by keeping coverage depth of at least 100X to increase the likelihood of each marker with a minimum coverage of 5X. The emphasis was given to design assays with high‐performing markers with overall mean marker call rates >90%. This assay was initially genotyped for 386 samples to check the quality of genotyping data, wherein many of the samples are being duplicated to check the consistency of call data. After ensuring high‐quality data, this newly developed genotyping assay was used for genotyping a set of 2,573 genotypes (270 FP of breeding programs [ICRISAT, UAS‐Dharwad, and ICAR‐DGR], 1506 ABLs, 297 RS, 294 TPs, and 206 RV). A distance matrix was constructed for each dataset, and MDS, similar to a PCA, was performed using TASSEL for all datasets (Figure 5). The first three principal components collectively explain 82.08% of the variance, with PC1 contributing 42.16%, PC2 explaining 24.56%, and PC3 explaining 15.36%, all indicating substantial variation among the genotypes. The results showed clear clustering of the RS, TP, and RV, while mixed for FP and ABLs. The genetic relationship information, along with genetic distance will help in selecting parents to sample the high genetic diversity for future breeding programs. Analysis of the PIC values for each set indicates that the markers are moderately informative, with PIC varying from 0.34 to 0.37. The proportion of heterozygosity across the different sets ranged from 0.027 to 0.294, with ABLs set 1 exhibited the lowest, whereas the RS showed the highest heterozygosity. Meanwhile, average minor allele frequency (MAF) values were calculated for each set, depicting the ABLs set 1 had the lowest MAF (0.062), while the RS had the highest (0.216). The calculated proportion of missing data for the six distinct sets showed that the RS had the lowest proportion (0.031), whereas the ABLs set 2 had the highest proportion (0.082) (Table 2). This information provides a significant understanding of the genetic diversity and variability within different sets, resulting in valuable clues for the characterization and selection of breeding lines.

**TABLE 2:** Summary of genetic diversity indices among distinct diverse sets of groundnut germplasm.

	Number of samples	Released varieties	Training population	Reference set	Founder parents	Advanced breeding lines set 2	Advanced breeding lines set 1
Number of samples	2573	206	294	297	270	805	701
PIC	0.32	0.34	0.34	0.36	0.36	0.37	0.35
Average minor allele frequency	0.192	0.076	0.102	0.216	0.066	0.064	0.062
Proportion missing	0.065	0.034	0.041	0.031	0.061	0.082	0.081
Proportion not missing	0.934	0.966	0.958	0.968	0.938	0.917	0.918
Proportion heterozygous	0.068	0.044	0.09	0.294	0.03	0.031	0.027

Abbreviation: PIC, polymorphism information content.

### 3.3 Background genome recovery using mid‐density assay versus high‐density array

**FIGURE 6:**
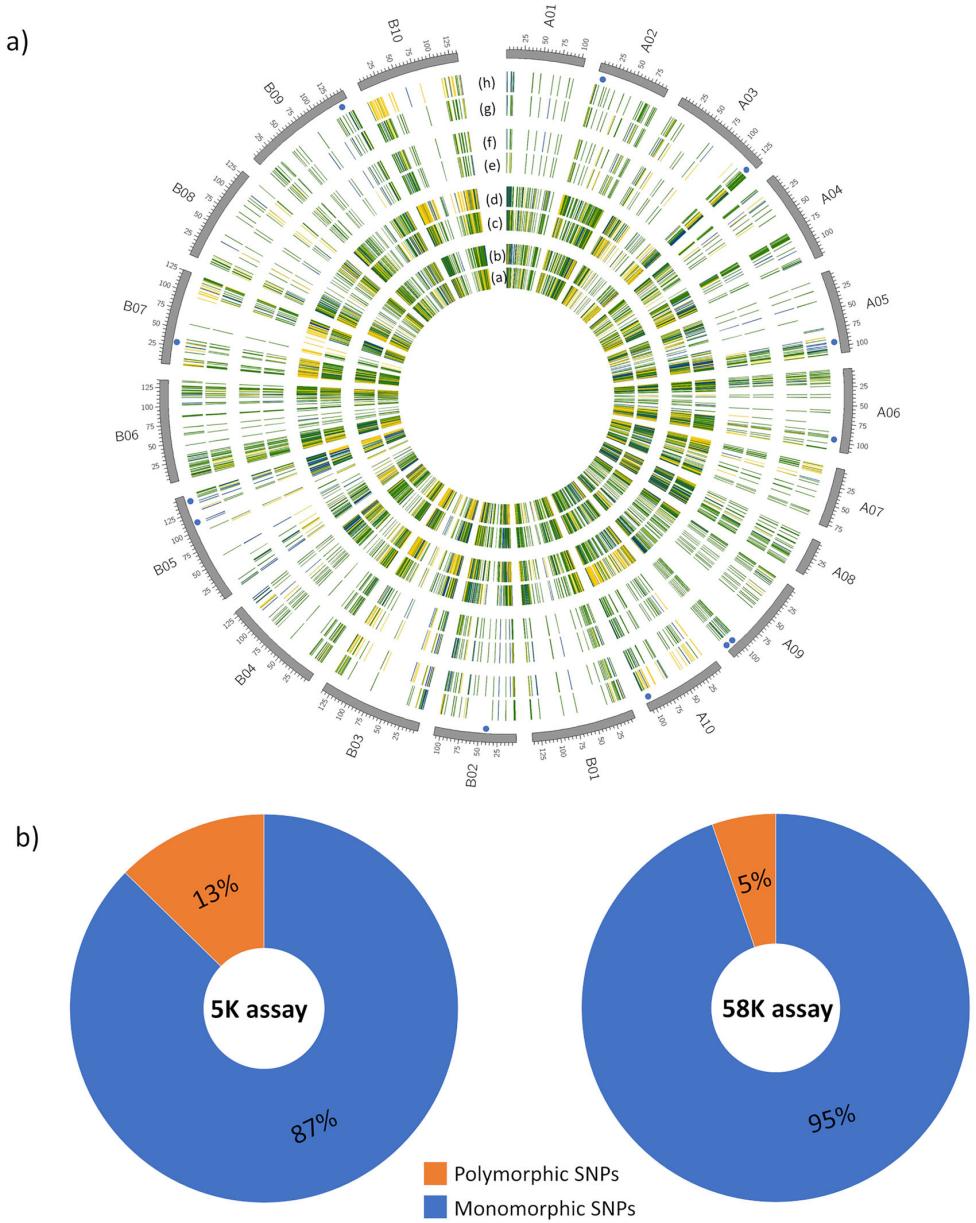
Background genome recovery using 58K array and 5K assay. (a) Circos plot illustrates background genome recovery of four marker‐assisted backcross lines using 58 and 5K assays. The tracks from inside to outside (a–d) illustrate background genome recovery of four marker‐assisted backcrossing (MABC) lines using 58K array, whereas tracks (e–h) illustrate background genome recovery of the same set of four MABC lines using 5K assay. Uniform whole genome recovery across 20 chromosomes of groundnut was achieved using 5K mid‐density assay. (b) Number of polymorphic single nucleotide polymorphisms (SNPs) recovered from 5K assay and 58K array. 13% in 5K assay and 5% in 58K array were identified as polymorphic SNPs between parents of MABC lines.

A set of four marker‐assisted backcrossing (MABC) lines in BC_3_F_4_ generation, derived from the cross GPBD4 and GJG9 were genotyped using the 58K high‐density array and 5K mid‐density assay to compare the background genome recovery (Figure 6a). GJG9 was recurrent parent (background genome) susceptible to late leaf spot and GPBD4 was used as donor for late leaf spot resistance. A total of 726 SNPs (13%) in 5K assay were polymorphic between parents GPBD4 and GJG9 of MABC lines, as compared to 2,900 SNPs (5%) in 58K array (Figure 6b). The background genome recovery was more densely visualized using a circos plot with a high‐density SNP array, in comparison to a mid‐density assay. Interestingly, uniform recovery of background genome was achieved from each of the 20 chromosomes using mid‐density assay. Therefore, mid‐density assay presents a cost‐effective option for background genome recovery in a large number of breeding populations.

## 4 DISCUSSION

Genomics advancements have facilitated the development of cost‐effective and highly precise SNP genotyping arrays. These arrays have proven important in accelerating breeding innovations and providing essential support for fundamental research in the area of crop breeding. In 2017, we previously developed high‐density genotyping array containing 58,233 unique and informative SNPs (Pandey et al., 2017). This high‐density SNP array “Axiom*_Arachis*” has been extensively used for performing diversity analysis (Clevenger et al., 2017), trait mapping (Dodia et al., 2019; Luo et al., 2019; Pandey et al., 2020), background selection in backcross breeding program (Shasidhar et al., 2020), and genomic prediction (Pandey et al., 2020) research in groundnut by multiple research groups. We have used this genotyping assay to genotype a total of 722 cultivated groundnut genotypes. After analyzing these data, we selected 5,000 SNPs using the criteria of PIC and uniform genome coverage. SNPs having PIC value of 0.374 and above were shortlisted. For the final assay design, 5,081 SNPs were selected. Such customized SNP assays, comprising SNPs chosen from sequencing data and past association research, offer a valuable genotyping platform due to its efficacy, accessibility, and precision (Morales et al., 2020; Sun et al., 2020; Tian et al., 2021).

The groundnut AgriSeq T‐GBS MDG assay has 5,081 SNP markers with balanced sub‐genome representation of 2,428 SNPs on the A sub‐genome (Chromosome 1–10) and 2,653 SNPs on the B sub‐genome (Chromosome 11–20). Of these, 82 SNPs have been associated with important traits such as rust and late leaf spot resistance, high oleic acid, seed weight, fresh seed dormancy, and shelling percentage for the purpose of foreground selection. Trait‐specific SNPs serve as effective tools in MAS, allowing breeders to find and select traits of interest more efficiently. Kompetitive Allele Specific polymerase chain reaction markers have been successfully employed, aiding in the selection of traits like disease resistance and quality enhancement (Mbanjo et al., 2024). The reliability of SNP marker predictions is significantly influenced by their association with the trait. Major‐effect SNPs linked to key genes often show higher prediction accuracy, while complex traits governed by multiple genes and influenced by environmental factors may result in lower accuracy (Heffner et al., 2009). SNP markers are especially valuable for traits that are difficult to evaluate phenotypically, such as drought tolerance or nutritional content. In such instances, trait‐specific SNP facilitates indirect selection without extensive and time‐consuming phenotypic evaluation (Xu & Crouch, 2008). Integrating validated SNP markers allows into MAS pipelines enables breeders to more effectively target traits of interest, pyramid favorable alleles, which can improve both the accuracy and success rate of breeding programs.

This study presents an investigation of molecular diversity by employing the developed panel on a diverse set of groundnut genotypes. The findings from the analysis of 2,573 genotypes provided support for the efficacy of the newly developed SNP assay in identifying genetic variation among genotypes with varied genomic constitutions. The assay facilitated an understanding of the genetic diversity present within various sets of groundnut collections, enabling a comprehensive assessment of genetic diversity indices. This will facilitate the identification of conserved alleles specific to sets of genotypes, as well as with potential implications in breeding and evolutionary research.

This assay, characterized by its cost‐effectiveness and faster data turnover, demonstrates great potential for routine applications as a MDG platform in groundnut research programs such as (a) checking genetic purity among FP of the breeding program, (b) checking genetic purity within and between accessions, including the identification of duplicate germplasm in genebank collections, (c) DNA fingerprinting of breeding material for legal validation, (d) tracing genetic linkage drag from wilds and distant relatives in wide crossing pre‐breeding programs, (e) facilitating background genome recovery in backcross breeding program, (f) enabling genomic prediction based selection strategies in genomic selection breeding, and (g) verifying genetic purity in RV in seed system.

Measures of genetic indices from the AgriSeq MDG assay provided insights into the diversity aspects of diverse sets of groundnut collection. Both the PIC values and the proportion of heterozygosity provide measures of genetic diversity among genotypes. These metrics offer insights into the evolutionary pressure on alleles and the mutation rate experienced by a locus through time. When evaluating the inheritance from parents to offspring by linkage analysis, the PIC values provide a reliable measure of the markers' efficacy (Shete et al., 2000). In contrast, heterozygosity serves as a means to determine genetic distance among individuals within a population, in addition to determining gene diversity for haploid markers. Using the AgriSeq MDG assay, the calculated average PIC values of markers within each group were less than 0.5 (PIC = 0.34–0.37), indicating a moderately informative. This level of PIC value is expected for SNP markers utilized in genotyping. For bi‐allelic SNPs and low mutation rates on SNP loci, the value is constrained to 0.5 (Eltaher et al., 2018). The MAF of each estimated diverse set also showed the difference among common and rare alleles in genotypes, resulting in MAF as an important factor in selection and evolution. The mean MAF from the inferred set comprising ABLs set 1 is 0.062 (relatively low), which means that the common alleles dominate most of the alleles in this set. This observation, however, is in contrast to findings in diverse RS where we observed the mean MAF of 0.216, indicating greater diversity in this set compared to the breeding set.

The advent of various genotyping technologies in recent years has made it simpler to perform SNP genotyping. The developed groundnut AgriSeq T‐GBS mid‐density assay fulfills the requirement for such SNP genotyping in groundnut more readily. This assay has facilitated a precise assessment of the structural complexity and genetic diversity in groundnut, holding importance for germplasm characterization, genomics studies, and marker‐assisted breeding. The diverse sets analyzed in this study, including RSs, FP, RV, TPs, and ABLs from stage 1 and stage 2 trials, contribute valuable insights into genetic variability and SNP profile, particularly for groundnut breeding. Researchers now have the opportunity to leverage these markers for investigating and examining the genetic diversity in germplasm from various origins and explore the genetic foundation of complex traits.

Moreover, two genetic mapping studies utilizing the AgriSeq T‐GBS mid‐density assay have revealed important findings for fresh seed dormancy and iron (Fe) and zinc (Zn) content traits in groundnut. The first study involved a recombinant inbred line ICGV 02266 × ICGV 97045 population, identified five major quantitative trait loci (QTLs) for fresh seed dormancy on five chromosomes (Ah01, Ah11, Ah06, Ah16, and Ah17). These QTLs explained phenotypic variance ranging from 53.2% to 60.4% (QTL Cartographer software) and 69.3% to 74.7% (IciMapping software). Gene mining in the vicinity of these genomic regions highlighted key regulators, like histone deacetylases and ethylene‐responsive transcription factors associated with hormonal regulation of dormancy (Bomireddy et al., 2022). In another study, utilizing a biparental mapping population (ICGV 00440 × ICGV 06040), five major main‐effect QTLs for Fe (explaining phenotypic variance ranging from 22% to 30.0%) and four major main‐effect QTLs for Zn content (explaining from 21.8% to 32.8% phenotypic variance) were identified (Parmar et al., 2023). High‐resolution QTL mapping requires high‐density marker arrays, which is limitation in our mid‐density assay. Due to the limited number of SNPs in assay, adoption in trait mapping would requires a high population size to provide the appropriate statistical accuracy for QTL detection (Wang et al., 2019). This mid‐density assay has notable potential for examining population structure, assessing genetic diversity, and helping for QC in the breeding program. Therefore, the extensive genomic SNP resources and fundamental knowledge generated from this research would establish a robust framework, thereby promoting swift advancements in groundnut genomic resources and facilitating the acceleration of groundnut breeding programs.

## 5 SUMMARY

A cost‐effective high‐throughput MDG assay is essential for large‐scale genomic breeding applications in addition to genetic purity and duplication detection in gene bank and genetic purity among the seed system. The newly developed mid‐density assay with 5,081 SNPs in groundnut provides new opportunities for deployment in these applications on a large scale. Being dynamic, AgriSeq T‐GBS assays can be easily modified by adding informative SNPs in the future to further increase the utilization of this assay in breeding programs globally.

## AUTHOR CONTRIBUTIONS

**Manish K. Pandey**: Conceptualization; data curation; formal analysis; funding acquisition; investigation; resources; writing—original draft; writing—review and editing. **Vinay Sharma**: Data curation; formal analysis; methodology; software; visualization; writing—original draft; writing—review and editing. **Aamir W. Khan**: Data curation; formal analysis; software. **Pushpesh Joshi**: Data curation; methodology. **Sunil S. Gangurde**: Data curation; formal analysis; methodology; software; writing—original draft; writing—review and editing. **Prasad Bajaj**: Formal analysis; methodology. **Pasupuleti Janila**: Investigation, resources. **Annapurna Chitikineni**: Investigation; methodology. **Ramesh Bhat**: Investigation, resources. **Babu N. Motagi**: Investigation, resources. **Chandramohan Sangh**: Investigation; resources. **Thankappan Radhakrishnan**: Investigation; resources; writing—review and editing. **Sandip K. Bera**: Investigation, resources. **Gregor Gorjanc**: Investigation; resources; writing—review and editing. **Krishna Reddy Gujjula**: Data curation, formal analysis. **Nathan Hall**: Data curation; formal analysis. **Claudio D. Carrasco**: Data curation, formal analysis. **Kandalam Arjun**: Data curation; formal analysis. **Srinivas Chandram**: Data curation, formal analysis. **Rajeev K. Varshney**: Conceptualization; investigation; resources; writing—original draft; writing—review and editing.

## CONFLICT OF INTEREST STATEMENT

The authors declare no conflicts of interest.

## Supporting information




**Table S1**: List of 722 genotypes used for genotyping using high density SNP array
**Table S2**: List of 8,027 SNPs selected based on PIC values
**Table S3**: List of 82 SNPs associated with various traits with allele information and flanking sequences
**Table S4**: List of 5,081 SNPs with details of chromosome number, position in diploid and tetraploid genome

## Data Availability

All the data generated in this study are provided in the supplementary material.
